# Imaging Tips to Recognize Primary Breast Angiosarcoma

**DOI:** 10.5334/jbsr.2629

**Published:** 2022-01-19

**Authors:** Patricia Costa, Anabela Ferrão, Lígia Pires-Gonçalves

**Affiliations:** 1 Hospital Pedro Hispano, PT; 2 Instituto Português de Oncologia do Porto, PT

**Keywords:** angiosarcoma, breast neoplasm, ultrasonography, Doppler, contrast-enhanced mammography, magnetic resonance imaging

## Abstract

**Teaching Point**: Primary breast angiosarcoma should be in the differential of a breast mass with rapid growth. It typically appears intensely vascularized and non-calcified, predominantly hyperechoic, and hyperintense on T2-weighted MRI.

## Case Study

A 55-year-old female presented to a primary care facility with an ill-defined lump in the right breast, near the site of a fibroadenoma’s lumpectomy performed 15 years earlier. The initial mammography and ultrasound were interpreted as postoperative fibrosis.

**Figure 1:**
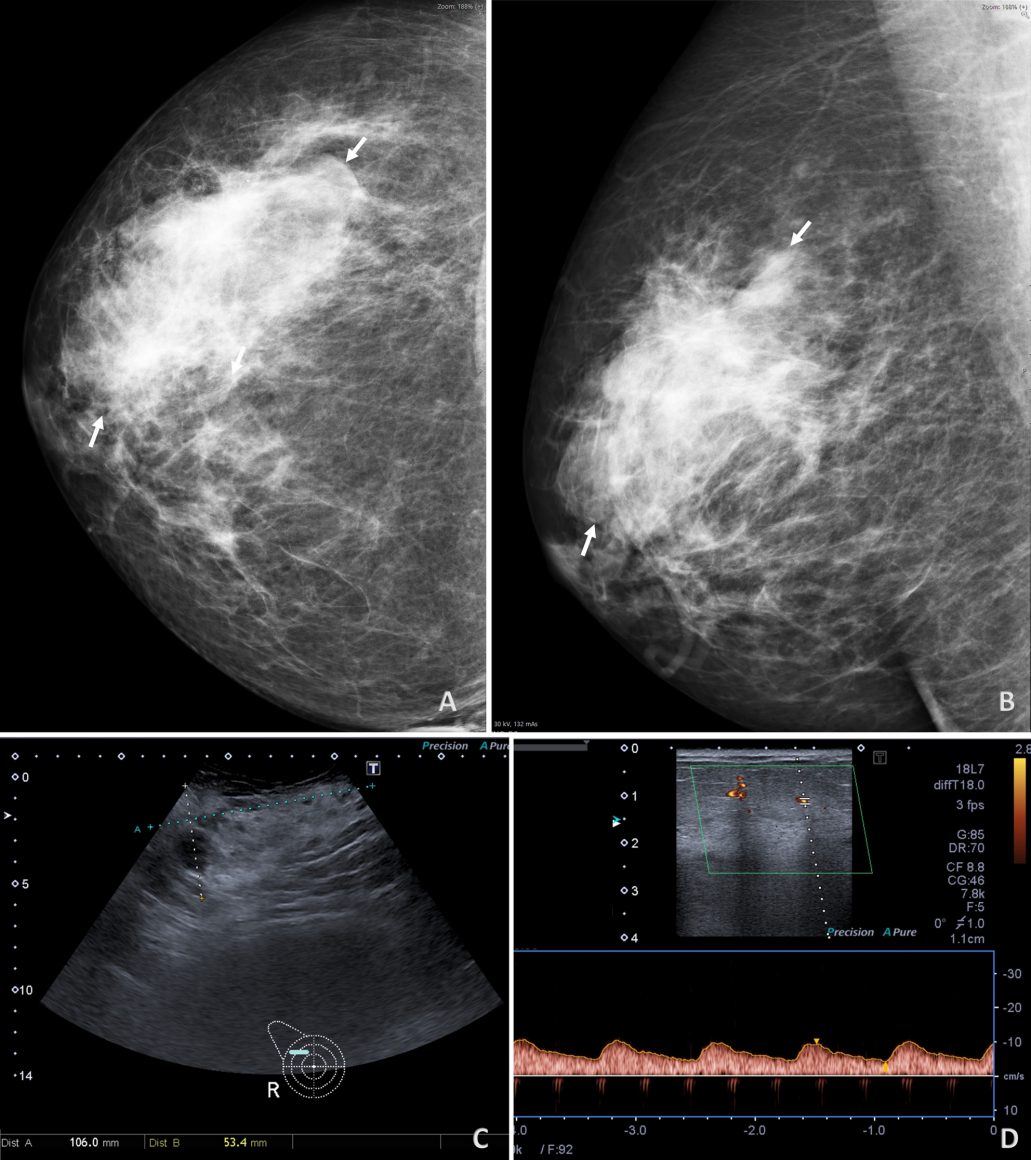


Eight months later, the patient was referred to our institution due to rapid growth (up to 10 cm) of the lump. Mammography revealed a non-calcified asymmetry (***Figure 1A–B***, arrows) corresponding on ultrasound to an ill-defined heterogeneous predominantly hyperechoic mass (***Figure 1C***). Spectral Doppler demonstrated intra-tumoral low-resistance arteriolar flow (***Figure 1D***).

**Figure 2:**
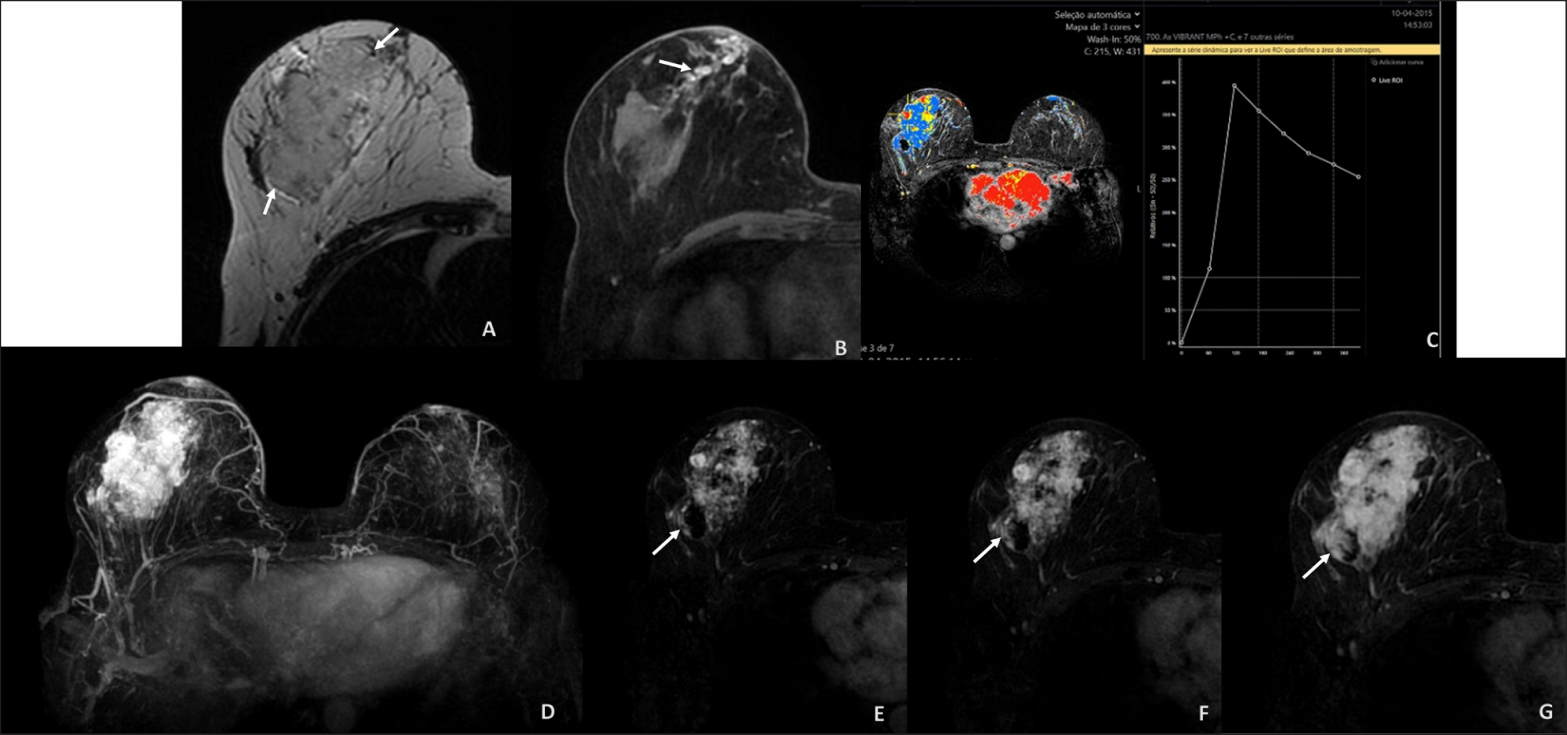


Magnetic resonance imaging (MRI) showed a T2-hyperintense large lobulated mass (arrows, ***Figure 2A***), with intra-ductal T1-hyperintense blood products (arrows, ***Figure 2B***) and heterogeneous enhancement, mixing areas of intense initial enhancement with washout (***Figure 2C***), typical of malignancy, with others of progressive centripetal filling (***Figure 2E–G***, arrows), suggesting a vascular lesion. There was neither axillary lymph node enlargement nor skin involvement.

Ultrasound-guided core biopsy confirmed the diagnosis of primary breast angiosarcoma (PBA). The patient underwent right total mastectomy and adjuvant radiotherapy.

**Figure 3:**
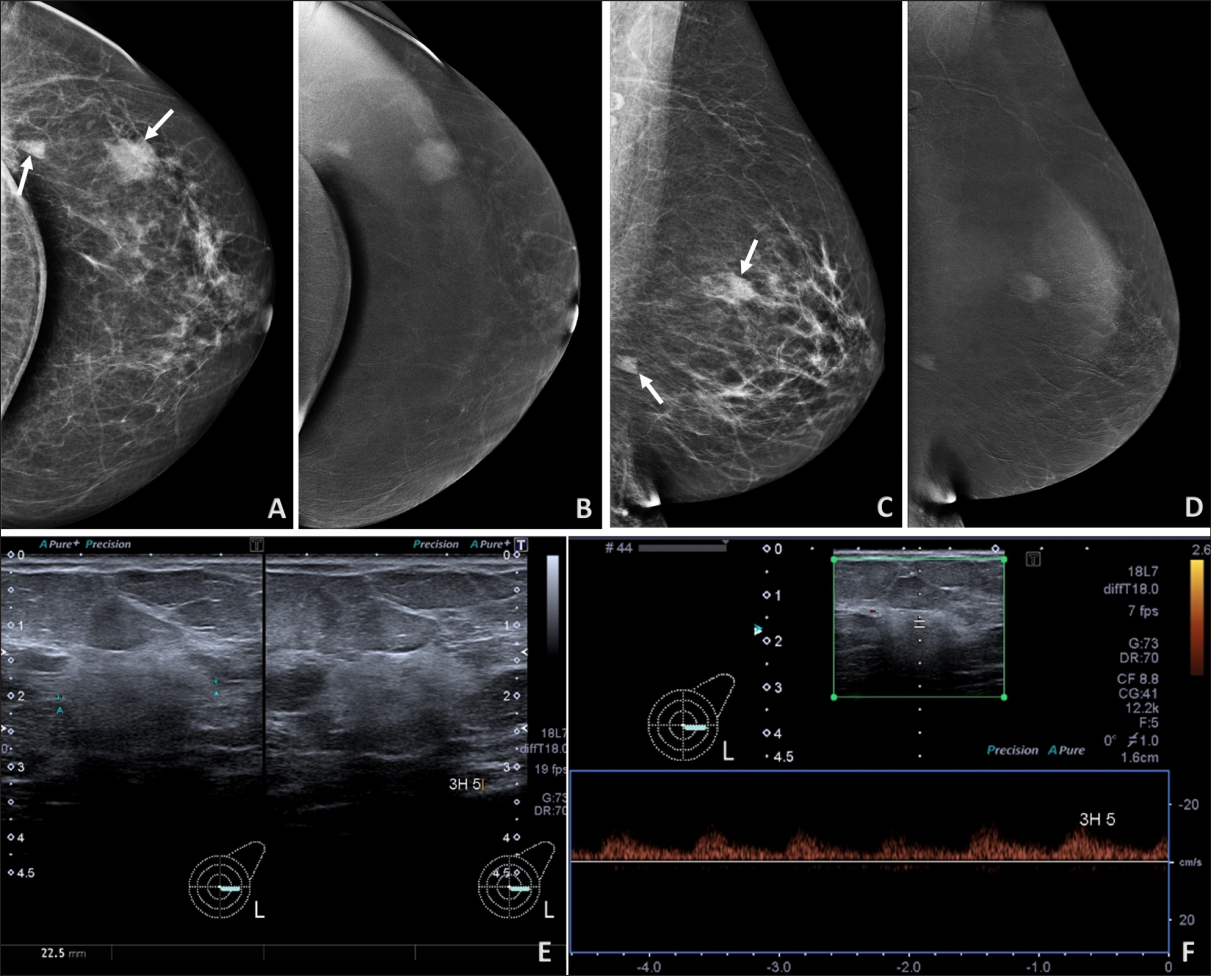


At the second year of follow-up, two left-sided non-calcified masses were identified on mammography (arrows, ***Figure 3A, C***), corresponding to ill-defined hyperechoic and vascularized nodules at ultrasound (***Figure 3E–F***). Contrast-enhanced mammography confirmed avid enhancement (***Figure 3B, D***), suggesting contralateral angiosarcoma, histologically confirmed.

## Comment

Breast angiosarcoma is rare and mostly secondary to radiotherapy. Although imaging is similar, the primary type is much rarer and presents at a younger age (mostly in the fourth and fifth decades), usually without skin involvement (a classic feature of the secondary form) [1].

PBA is frequently symptomatic at diagnosis, given its rapid growth and presentation before the recommended age for mammographic screening [1].

Mammography usually identifies a non-calcified ill-defined mass or focal or developing asymmetry, although it can be unrevealing [1].

Ultrasound frequently demonstrates a mixed hyper-and hypoechoic ill-defined lesion, previously reported as relatively specific of PBA and most common in larger lesions [1]. Angiosarcoma can also present as a hypo-or hyperechoic mass, the latter being most often associated with benign entities such as fat-containing lesions and fibrosis. Doppler can be decisive in this setting, as it identifies low-resitance flow [1].

MRI is valuable for both diagnosis and staging. It might distinguish fibrotic from neoplastic tissue, which may have been useful in our patient’s first presentation. PBA is typically a large heterogeneous mass with indistinct borders, T2-hyperintense (typical of vascular lesions), with areas of high T1-signal, in keeping with haemorrhage. Enhancement is usually intense and heterogeneous with washout [1].

Contrast-enhanced mammography can demonstrate breast vascularity and is cheaper, better tolerated, and more available than MRI. We herein reported for the first time that the hypervascularity of PBA could be depicted by contrast-enhanced mammography.

## References

[1] Yang WT, Hennessy BTJ, Dryden MJ, Valero V, Hunt KK, Krishnamurthy S. Mammary angiosarcomas: Imaging findings in 24 patients. Radiology. 2007 Mar; 242(3): 725–34. DOI: 10.1148/radiol.2423060163 17325063

